# Possibility of High Ionic Conductivity and High Fracture Toughness in All-Dislocation-Ceramics

**DOI:** 10.3390/ma17020428

**Published:** 2024-01-15

**Authors:** Kyuichi Yasui, Koichi Hamamoto

**Affiliations:** National Institute of Advanced Industrial Science and Technology (AIST), Nagoya 463-8560, Japan; k-hamamoto@aist.go.jp

**Keywords:** dislocation, solid electrolyte, ionic conductivity, dendrite, fracture toughness, all-solid-state batteries

## Abstract

Based on the results of numerical calculations as well as those of some related experiments which are reviewed in the present paper, it is suggested that solid electrolytes filled with appropriate dislocations, which is called all-dislocation-ceramics, are expected to have considerably higher ionic conductivity and higher fracture toughness than those of normal solid electrolytes. Higher ionic conductivity is due to the huge ionic conductivity along dislocations where the formation energy of vacancies is considerably lower than that in the bulk solid. Furthermore, in all-dislocation- ceramics, dendrite formation could be avoided. Higher fracture toughness is due to enhanced emissions of dislocations from a crack tip by pre-existing dislocations, which causes shielding of a crack tip, energy dissipation due to plastic deformation and heating, and crack-tip blunting. All-dislocation-ceramics may be useful for all-solid-state batteries.

## 1. Introduction

Lithium-ion batteries with flammable organic electrolytes have some safety problems due to their ignition capability from overcharging or short-circuiting, etc. [1]. In order to solve the safety problem, all-solid-state batteries have been intensively studied in recent years because they are nonflammable and there is no liquid leakage [1,2,3,4,5]. However, ionic conductivity in solid electrolytes is generally lower than that in liquid electrolytes [2,6,7,8]. It is required to increase ionic conductivity in solid electrolytes to achieve higher power densities of all-solid-state batteries with higher charge/discharge rates, as well as larger dimensions of a battery with lower internal resistance [5,9,10,11]. In addition, for applications of solid electrolytes to all-solid-state batteries, appropriate mechanical properties of solid electrolytes are required. To prevent the creation and propagation of fractures by electrochemical shock, which deteriorate the battery performance significantly, and to prevent dendrite formation from crack propagation, which short circuits the battery, high fracture toughness is required for solid electrolytes [12,13,14,15,16].

In the present review, the possibility of high ionic conductivity and high fracture toughness by introducing appropriate dislocations into solid electrolytes is discussed based on the results of numerical calculations, as well as some related experiments. We have already suggested theoretically that ionic conductivity could be increased by several orders of magnitude by filling solid electrolytes with appropriate dislocations without dendrite formation [17,18]. We call such solid electrolytes all-dislocation-ceramics. Some researchers in other research groups [19,20,21] have experimentally shown that the introduction of dislocations could increase the fracture toughness of ceramics. In the present review, the mechanism of the toughening of ceramics by the introduction of dislocations is discussed in detail. When a ceramic specimen is filled with dislocations, the strength of the specimen may become considerably lower. Nevertheless, fracture toughness, which is the resistance to crack propagation, could be increased by the presence of dislocations because pre-existing dislocations enhance emissions of new dislocations from a crack tip, which shield the crack tip by inducing compressive stress. In addition, the emissions of dislocations from a crack tip dissipate some energy through plastic deformation and heating, as well as blunting of the crack tip, which further increases the fracture toughness of the specimen. The aim of the present review is to discuss the possibility of the application of all-dislocation-ceramics to all-solid-state batteries due to their high ionic conductivity and high fracture toughness.

## 2. High Ionic Conductivity without Dendrite Formation

### 2.1. High Ionic Conductivity in All-Dislocation-Ceramics

In the present subsection, a theoretical study [17] on ionic conductivity in single-crystal ceramics filled with parallel straight dislocations is reviewed. The theoretical study is based on the experimental observation [22,23,24,25,26,27] that ionic conductivity along dislocations is several orders of magnitude higher than that in the bulk. Higher ionic conductivity is also suggested by a higher diffusion coefficient along dislocations according to the Nernst–Einstein equation [17,28]. The reason for the extremely high ionic conductivity along dislocations is the much lower formation energy of vacancies along dislocations compared to that in the bulk [23,24,26,29,30,31,32]. The region around a dislocation with extremely high ionic conductivity is called a dislocation pipe. The diameter of a dislocation pipe (δ) is determined by the spatial distribution of the space charge region in which charged vacancies and interstitials are gathered around an oppositely charged dislocation core [26,33,34,35]. The diameter of a dislocation pipe (δ) is, however, much smaller than that of the space charge region and ranges from 0.2 to 3 nm [27,31,36,37].

Recently, improvement in the functional and electrical properties of ceramics by the introduction of dislocations has been intensively studied, which is called dislocation engineering [19,20,21,24,33,38,39,40,41]. In many cases, the introduction of dislocations into ceramics is by applying compressive stress to a specimen at room temperature or elevated temperatures [24,33,38,39,40,42,43,44,45,46,47,48,49,50]. There are also other methods such as mechanical polishing [19,51], cyclic loading of a stainless steel indenter [52], etc. The typical density of dislocations introduced into ceramics ranges from $10^{12}$ to $10^{16}$ $m^{-2}$, while the initial value is about $10^{9}$ to $10^{10}$ $m^{-2}$ [45,48,49,51,52,53].

**Figure 1:**
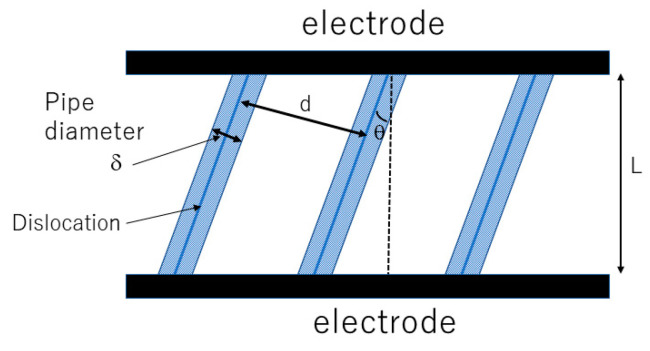
Model of single-crystal electrolyte with parallel straight dislocations. Reprinted with permission from Ref. [17]. Copyright 2023, IOP Publishing Ltd.

In the theoretical analysis [17], parallel straight dislocations are considered because such dislocations are often experimentally observed [35,39,40,45,49,54]. A model of a solid electrolyte with parallel straight dislocations is shown in Figure 1 [17]. In the theoretical analysis [17], not only ionic conductivity but also dendrite formation is studied because even in solid electrolytes dendrite formation is one of the severe problems [55,56,57,58,59,60]. The ionic current is concentrated along dislocations, and dendrites could be developed from the electrode along dislocations [61]. The possibility of dendrite formation is theoretically studied by numerical calculations of the spatial variation of ionic conductivity [17].

The distance between neighboring dislocations (d) in Figure 1 is given as follows.

(1) $$d=\frac{1}{\sqrt{n_{d}}}$$

where $n_{d}$ is the density of dislocations. The mean ionic conductivity (κ) of the single-crystal solid electrolyte in Figure 1 is approximately given as follows [17].

When δ<d, where δ is the diameter of a dislocation pipe,

(2) $$\kappa =\kappa_{d}{\left(\cos\theta \right)}^{2}n_{d}S_{d}+\kappa_{b}{\left(\sin\theta \right)}^{2}n_{d}S_{d}+\kappa_{b}\left(1-n_{d}S_{d}\right)-A\left(1-n_{d}S_{d}\right)$$

When $\delta \geq \delta_{c}$, where $\delta_{c}$ is the critical diameter of a dislocation pipe and this condition means that the single-crystal solid electrolyte is filled with dislocation pipes,

(3) $$\kappa =\kappa_{d}{\left(\cos\theta \right)}^{2}+\kappa_{d⊥}{\left(\sin\theta \right)}^{2}$$

where $\kappa_{d}$ and $\kappa_{b}$ are ionic conductivity along a dislocation and that in the bulk, respectively. $\kappa_{d⊥}$ is ionic conductivity across a dislocation which is much lower than that in the bulk [62,63,64]. In the theoretical analysis [17], $\frac{\kappa_{d⊥}}{\kappa_{b}}=10^{-2}$ is assumed. $\frac{\kappa_{d}}{\kappa_{b}}=10^{7}$ is assumed because ionic conductivity along a dislocation has been experimentally reported to be 5–7 orders of magnitude higher than that in the bulk [22,23,27]. δ=3 nm is assumed [36]. In Equation (2), $S_{d}$ is the cross section of a dislocation pipe, and A is the amplitude of reduction in ionic conductivity by crossing dislocations. The first term in the right side of Equation (2) is the contribution of ionic conduction along dislocations. The second term is the contribution of ionic conduction leaking from dislocations. The third term is the contribution of ionic conduction in the bulk. The last term is the reduction in ionic conductivity by crossing dislocations. The first term in the right side of Equation (3) is the contribution of ionic conduction along dislocations. The second term is that crossing dislocations. For more details of the theoretical analysis, please see Ref [17].

The spatial variation of ionic current density on the opposite electrode is crudely estimated as follows to study the possibility of dendrite formation because concentrations of ionic current density along dislocations could result in dendrite formation, as already discussed.

When δ<d,

(4) $$j_{dis}=\kappa_{d}E_{el}{\left(\cos\theta \right)}^{2}$$

(5) $$j_{other}=\kappa_{b}E_{el}+\kappa_{b}E_{el}{\left(\sin\theta \right)}^{2}\left(\frac{n_{d}S_{d}}{1-n_{d}S_{d}}\right)-AE_{el}$$

When $\delta \geq \delta_{c}$,

(6) $$\frac{j_{dis}}{j_{other}}=1$$

where $j_{dis}$ and $j_{other}$ are the ionic current density on a dislocation and that on the other area, respectively, on the opposite electrode. $E_{el}$ is the applied electric field. When $\delta \geq \delta_{c}$, all the area is completely covered with dislocation pipes, which is the condition for all-dislocation-ceramics.

**Figure 2:**
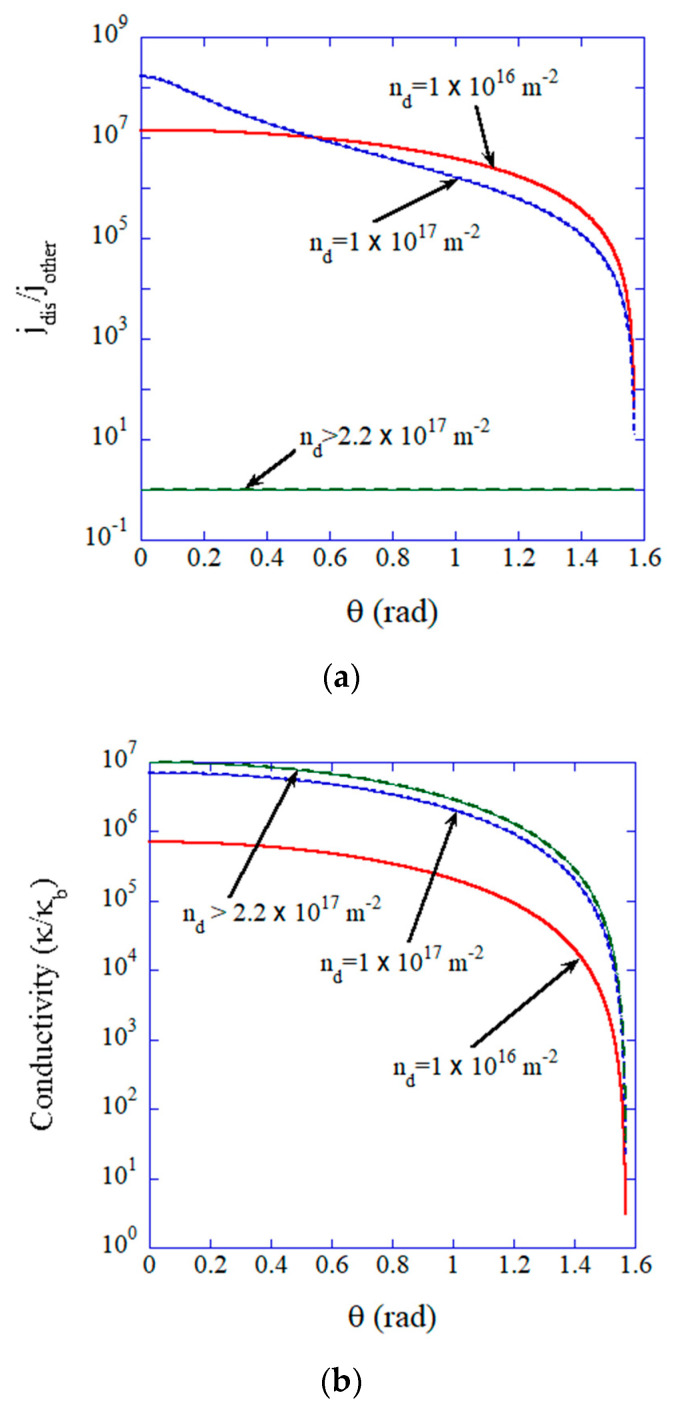
Influence of the dislocation density ($n_{d}$). (**a**) $j_{dis}/j_{other}$, where $j_{dis}$ and $j_{other}$ are the ionic current on a dislocation and that on the other area, respectively, on the opposite electrode, as a function of angle (θ) of the parallel dislocations. (**b**) The mean ionic conductivity ($\kappa /\kappa_{b}$) where $\kappa_{b}$ is ionic conductivity in the bulk. Reprinted with permission from Ref. [17]. Copyright 2023, IOP Publishing Ltd.

The results of the numerical calculations of Equations (2)–(6) are shown in Figure 2. When the dislocation density is higher than $2.2\times 10^{17}$ $m^{-2}$, which is the condition for all-dislocation-ceramics, there is no spatial variation of ionic current density on the opposite electrode and dendrite formation could be avoided (Figure 2a). Furthermore, the ionic conductivity of all-dislocation-ceramics is 5–7 orders of magnitude larger than that of normal solid electrolytes when the dislocations are nearly perpendicular to the electrodes (Figure 2b).

### 2.2. Theoretical Upper Limit of Dislocation Density in Ceramics

In order to study theoretically whether it is possible to produce all-dislocation-ceramics without fracture, the theoretical upper limit of dislocation density is investigated [18]. In the theoretical analysis, a simple model and probability model are constructed, in which a single value and a distribution of pre-existing microcrack size are assumed, respectively. Furthermore, a crystallographic limitation to avoid the transformation into the amorphous state and to avoid void formation are theoretically studied. In the simple and probability models, single-crystal ceramics is investigated because in polycrystals there are some additional complexities such as crack formation from pileup of dislocations at grain boundaries, etc. [65,66,67,68]. Furthermore, slight ductility of ceramics is assumed such that the Griffith criterion for brittle fracture and the Bailey–Hirsch type relationship between applied stress and the dislocation density are nearly valid simultaneously [18,65,69,70].

Firstly, the simple model is discussed [18]. According to the Griffith criterion for brittle fracture, the tensile strength ($\sigma_{t}$) of a solid specimen is given as follows [65,71].

(7) $$\left|\sigma_{t}\right|=\sqrt{\frac{\pi E\gamma_{s}}{\left(1-\nu^{2}\right)d_{crack}}}$$

where E is Young’s modulus, $\gamma_{s}$ is the surface energy of the solid specimen, ν is the Poisson’s ratio, and $d_{crack}$ is the diameter of a pre-existing penny-shaped (circular) mi-crocrack. In the theoretical analysis [18], the compressive strength is considered because in many cases compressive stress is applied to the specimen to introduce dislocations, as already noted. The compressive strength ($\sigma_{c}$) is crudely related to the tensile strength ($\sigma_{t}$), as follows [72].

(8) $$\sigma_{c}=R\left|\sigma_{t}\right|$$

where R is the strength ratio ranging from 2 to 64 [72,73]. The dislocation density ($n_{d}$) is assumed to be related to the magnitude of the applied compressive stress, (σ) like the Bai-ley–Hirsch relationship, as follows [69,70].

(9) $$\sigma \approx \alpha Gb\sqrt{n_{d}}$$

where α is a numerical factor of the order of 0.3, G is the shear modulus, and b is the magnitude of the Burgers vector. Although in the Bailey–Hirsch relationship applied stress is shear, compressive stress is assumed in Equation (9) because the plastic deformation under compressive stress is the cumulative effect of a large number of shear events which are sequential slips on various crystal planes [69]. From Equations (7)–(9), the condition for fracture during the application of compressive stress to introduce dislocations in ceramics is given as follows.

(10) $$\alpha Gb\sqrt{n_{d}}\geq R\times \sqrt{\frac{\pi E\gamma_{s}}{\left(1-\nu^{2}\right)d_{crack}}}\;$$

Accordingly, the upper limit of the dislocation density ($n_{d,limit}$) is approximately given as follows.

(11) $$n_{d,limit}\approx \frac{R^{2}}{{\left(\alpha Gb\right)}^{2}}\left(\frac{\pi E\gamma_{s}}{\left(1-\nu^{2}\right)d_{crack}}\right)$$

Next, the probability model is discussed [18]. When there is size distribution of pre-existing microcracks, the actual condition for fracture given by Equation (10) could be different for different specimens because the maximum size of pre-existing microcracks, which determines the compressive strength, could be different. In other words, the compressive strength as well as the upper limit of the dislocation density could be different for different specimens. Accordingly, the occurrence of fracture is expressed by a probability, which is the probability model. When $g\left(D\right)dD$ is the number concentration of pre-existing microcracks with a diameter between D and D+dD, the following relationship may hold according to the Weibull distribution [74,75,76].

(12) $$\int_{d_{c}}^{\infty }g\left(D\right)dD=n\times e^{\left[-{\left(\frac{d_{c}}{d_{0}}\right)}^{m}\right]}$$

where $d_{c}$ is the critical diameter of a pre-existing microcrack for fracture calculated by Equation (11), n is the number concentration of pre-existing microcracks, $d_{0}$ is the characteristic diameter of pre-existing microcracks, and m=1.192, which is the shape factor of the Weibull distribution, is employed [74]. Using Equation (12), the probability of frac-ture $P_{F}\left(V\right)$ in volume V of the specimen is derived as follows, according to Ref. [18].

(13) $$P_{F}=1-e^{\left[-Ne^{\left(-{\left(\frac{d_{c}}{d_{0}}\right)}^{m}\right)}\right]}$$

where N is the number of pre-existing microcracks in the specimen (N=nV).

Finally, the crystallographic limitation to avoid transformation into the amorphous state and that to avoid void formation are described. They are expressed by Inequalities (14) and (15), respectively [18].

(14) $$\frac{1}{\sqrt{n_{d}}}\gg b$$

(15) $$\frac{1}{\sqrt{n_{d}}}\geq \delta_{core}$$

where $\delta_{core}$ is the diameter of a dislocation core. If b=0.25 nm [6,77] and $\delta_{core}=1\;$nm [31] are assumed, Inequalities (14) and (15) are as follows.

(16) $$n_{d}\ll 1.6\times 10^{19}m^{-2}$$

(17) $$n_{d}\leq 10^{18}\;m^{-2}$$

In other words, the crystallographic limitation of dislocation density would be $10^{18}\;m^{-2}$.

**Figure 3:**
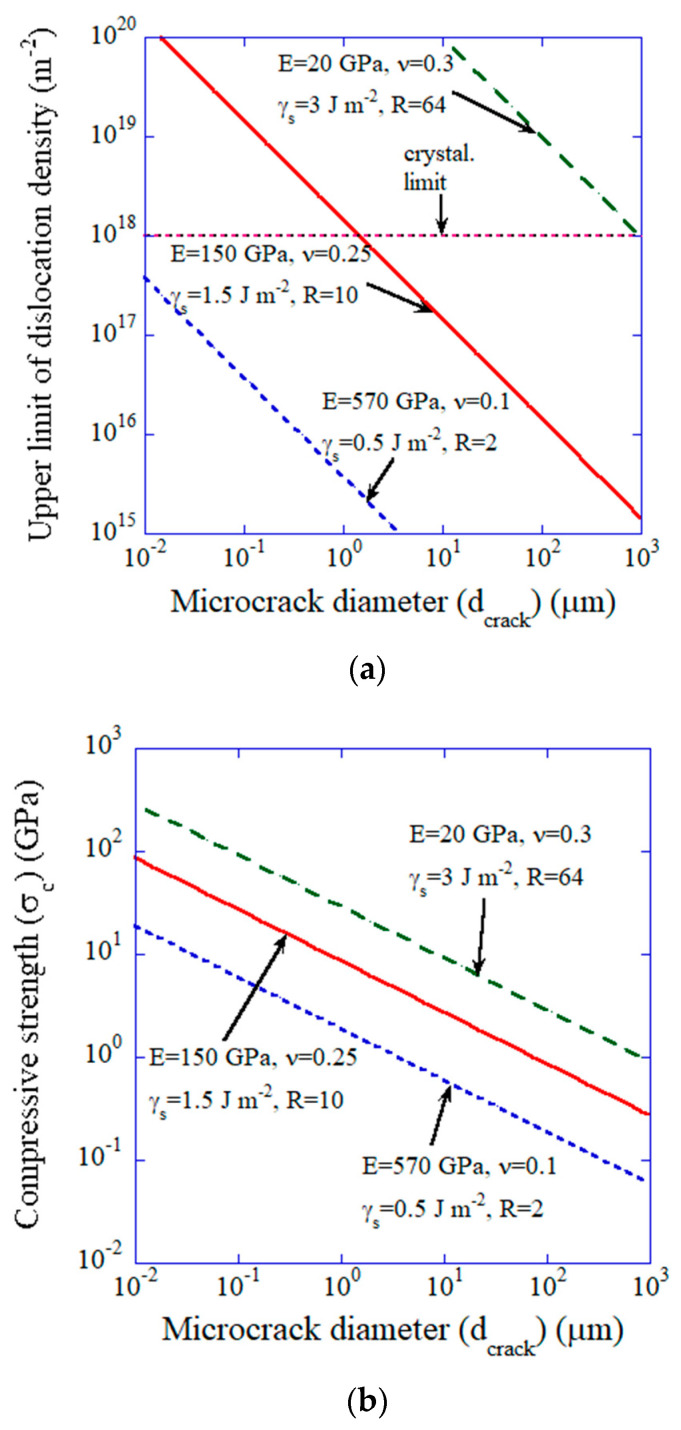
The results of numerical calculations for the simple model. (**a**) Upper limit of dislocation density ($\rho_{limit}$) as a function of microcrack diameter ($d_{crack}$). The crystallographic limit is also shown by red dotted line. (**b**) Compressive strength ($\sigma_{c})$ as a function of microcrack diameter ($d_{crack}$). Reprinted with permission from Ref. [18]. Copyright 2023, IOP Publishing Ltd.

The results of numerical calculations for the simple model are shown in Figure 3. In Figure 3a, the upper limit of dislocation density given by Equation (11) is shown as a function of microcrack diameter. The green dashed line and the blue dotted line are the upper and lower limit of the curve, respectively, for ceramics because the range of Young’s modulus is 20–570 GPa [78,79], that of the Poisson’s ratio is 0.10–0.30 [78], and that of the surface energy is 0.5–3 J·$m^{-2}$ [80,81]. The dislocation density ($2.2\times 10^{17}$ $m^{-2}$) required for all-dislocation-ceramics discussed in the previous subsection is achievable if the size of the pre-existing microcrack is sufficiently small (less than about 15 µm for the typical red curve in Figure 3a). Under those conditions, the compressive strength is higher than about 1 GPa, which would be sufficient for introducing dislocations into a ceramic specimen (Figure 3b).

**Figure 4:**
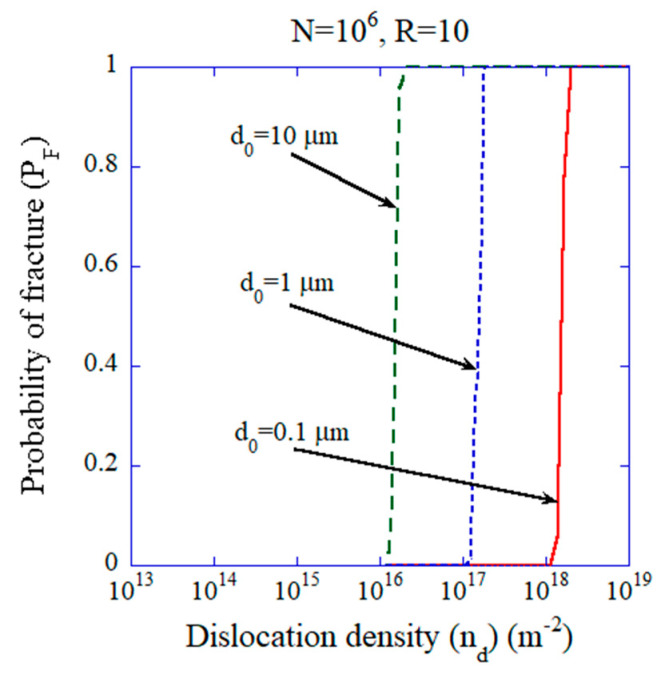
The results of numerical calculations for probability of the fracture ($P_{F}$) as a function of dislocation density when the number of microcracks is $N=10^{6}$ for various values of $d_{0}$ (the probability model). Reprinted with permission from Ref. [18]. Copyright 2023, IOP Publishing Ltd.

In Figure 4, the results of the numerical calculations for the probability model are shown on the probability of fracture given by Equation (13) as a function of dislocation density for $N=10^{6}$. The dislocation density ($2.2\times 10^{17}$ $m^{-2}$) required for all-dislocation- ceramics would be achievable if the typical diameter of the pre-existing microcrack is less than about 1 µm in this case, because there is some possibility for the existence of larger microcracks in the probability model.

## 3. Mechanism of Toughening by Pre-Existing Dislocations

### 3.1. Fracture Toughness

**Figure 5:**
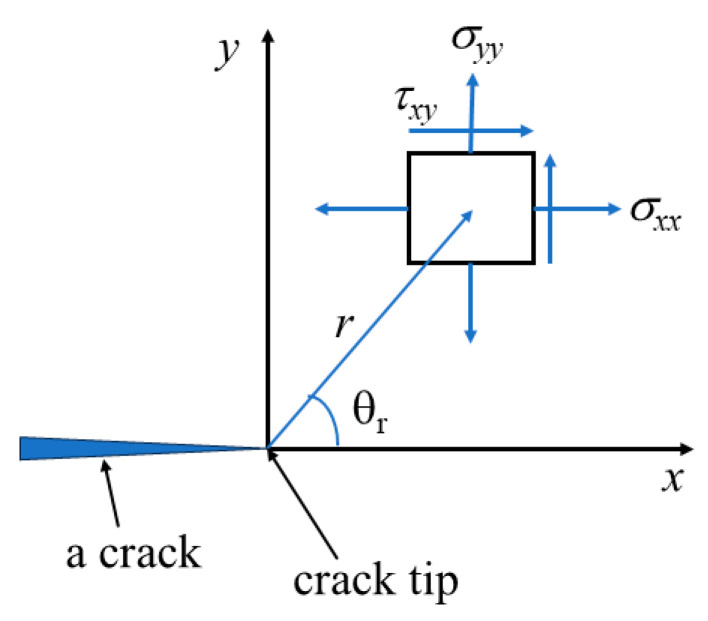
Stress filed around a crack tip. The infinitesimal cubic at distance *r* from the crack tip is exaggerated to clearly show the meaning of $\sigma_{xx}$, $\sigma_{yy}$, and $\tau_{xy}$.

In materials engineering, fracture toughness is an important parameter which characterizes resistance of the material to crack propagation [65,82,83]. In the actual measurement of fracture toughness, a relatively large crack (a notch) is made on a specimen, and the applied stress at the failure of the specimen is measured or the opening of the pre-existing crack under applied stress is measured, etc. [19,20,21,65,82,83,84,85]. Fracture toughness is defined as the critical stress intensity factor for crack propagation [65,82,83]. The stress intensity factor ($K_{I}$) is defined by the following equations to describe the stress field around a crack tip in an elastic material (Figure 5) [65,84].

(18) $$\sigma_{xx}=\frac{K_{I}}{\sqrt{2\pi r}}\cos\left(\frac{\theta_{r}}{2}\right)\left[1-\sin\left(\frac{\theta_{r}}{2}\right)\sin\left(\frac{3\theta_{r}}{2}\right)\right]$$

(19) $$\sigma_{yy}=\frac{K_{I}}{\sqrt{2\pi r}}\cos\left(\frac{\theta_{r}}{2}\right)\left[1+\sin\left(\frac{\theta_{r}}{2}\right)\sin\left(\frac{3\theta_{r}}{2}\right)\right]$$

(20) $$\tau_{xy}=\frac{K_{I}}{\sqrt{2\pi r}}\cos\left(\frac{\theta_{r}}{2}\right)\sin\left(\frac{\theta_{r}}{2}\right)\cos\left(\frac{3\theta_{r}}{2}\right)$$

where $\sigma_{xx}$, $\sigma_{yy}$, and $\tau_{xy}$ are stress components defined in Figure 5, r is the distance from a crack tip, and $\theta_{r}$ is the angle defined in Figure 5. In Equations (18)–(20), higher-order terms are neglected because only the stress field very close to the crack tip is considered [84]. The stress intensity factor ($K_{I}$) is expressed as follows [83].

(21) $$K_{I}=\sigma_{app}\sqrt{\pi aB}$$

where $\sigma_{app}$ is the magnitude of the applied stress on the specimen, a is the crack length, and B is a dimensionless factor which depends on the specimen geometry. When the stress intensity factor is higher than the fracture toughness ($K_{Ic}$), crack propagation occurs. The fracture toughness as well as stress intensity factor have dimensions of $MPa\cdot m^{1/2}$, according to Equations (18)–(20) [82]. According to a simple theoretical analysis [82], fracture toughness ($K_{Ic}$) is approximately expressed as follows.

(22) $$K_{Ic}\approx \sqrt{2\gamma_{s}E}$$

In other words, fracture toughness is approximately expressed only by material parameters, which is one of the reasons why fracture toughness has been widely used in material engineering. The fracture toughness of ceramics typically ranges from 0.2 to 20 $MPa\cdot m^{1/2}$ [82]. For metals, it ranges from 3 to 150 $MPa\cdot m^{1/2}$, which is typically higher than that of ceramics [86]. Failure of a specimen occurs under smaller magnitudes of the applied stress in the presence of a pre-existing large crack (notch) compared to a specimen without it. The critical stress for the former corresponds to the fracture toughness through Equation (21), while that for the latter is the strength of a specimen. The fracture toughness represents the resistance to propagation of a pre-existing large crack, while strength represents the resistance to failure without any pre-existing large crack. The fracture toughness is higher when the work by the applied stress is mostly used in the plastic deformation around the crack tip due to the generation and motion of dislocations from a crack tip, which is the case for most metals [82]. On the other hand, for most ceramics, crack-tip plasticity is much less than that in most metals, and the fracture toughness is mostly much lower [82]. The fracture toughness of ceramic solid-electrolytes is relatively low (~$1\;MPa\cdot m^{1/2}$), which is a major limitation in their mechanical properties [14,15,87,88].

**Figure 6:**
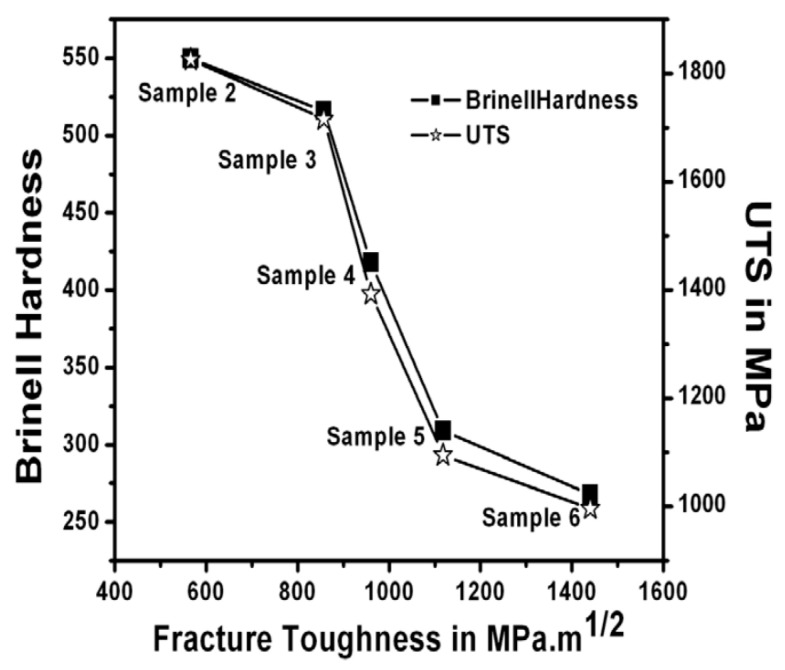
Correlation of fracture toughness with tensile strength (UTS: ultimate tensile strength) and hardness for engineering structural steel with various microstructures generated through heat treatment. Reprinted with permission from Ref. [89]. Copyright 2010, Springer Nature.

**Figure 7:**
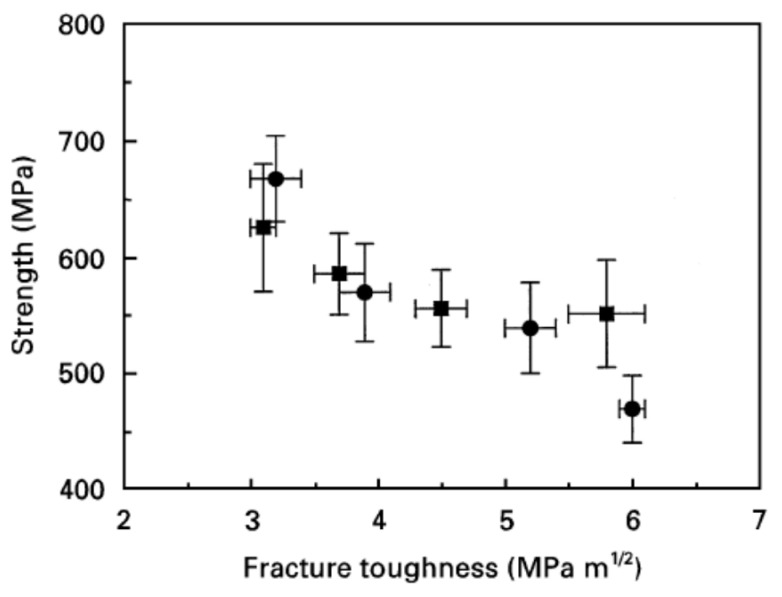
Relationship between flexural strength and fracture toughness of hot-pressed and annealed SiC ceramics. ■: SiC without α SiC seeds, ●: SiC with α SiC seeds. Reprinted with permission from Ref. [91]. Copyright 1997, Springer Nature.

**Figure 8:**
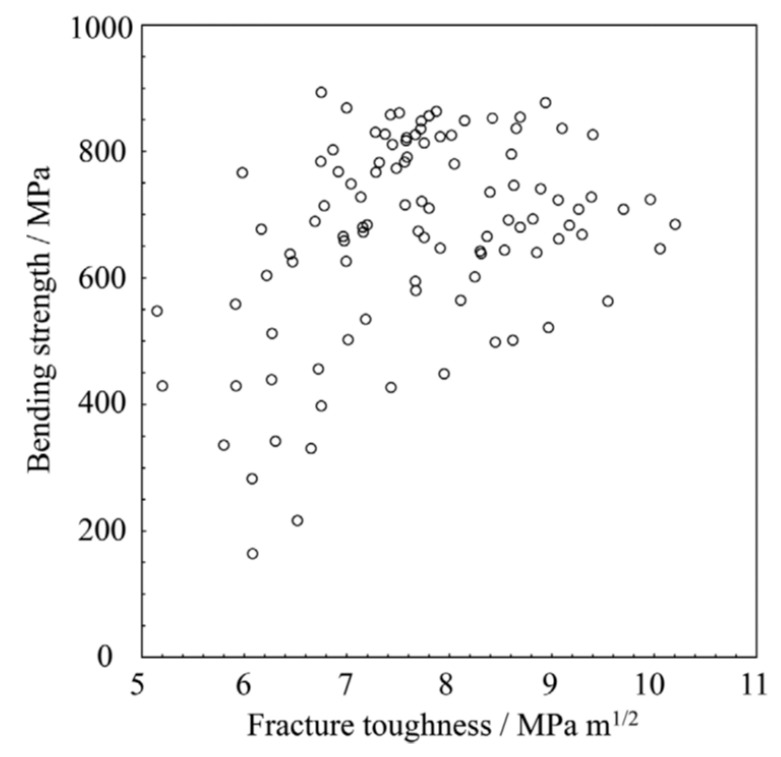
Relationship between flexural (bending) strength and fracture toughness of Si_3_N_4_ ceramics samples. Reprinted with permission from Ref. [92]. Copyright 2023, The American Ceramic Society, John Wiley.

Another reason why fracture toughness has been widely used is that the resistance to crack propagation is more important in engineering to avoid failure than the (tensile, compressive or flexural) strength of material. For many metals, fracture toughness is inversely related to the strength of the material (Figure 6) [89,90]. For ceramics, fracture toughness is sometimes inversely related to strength, but for other cases it is positively related to strength (Figure 7 and Figure 8) [91,92,93]. To enhance the fracture toughness of ceramics, a variety of approaches have been used. The essential idea is to increase the energy needed to extend a crack [82]. The basic approaches are crack deflection, crack bridging, and transformation toughening. Crack deflection occurs when a crack propagates along grain boundaries, which are generally declined relative to the initial direction of the crack propagation [82]. Indeed, the fracture toughness of polycrystalline alumina (about 4 MPa·$m^{1/2}$) is much higher than that of single-crystal alumina (about 2.2 MPa·$m^{1/2}$) [82]. Crack bifurcation around grains is also another reason for the higher fracture toughness of polycrystalline ceramics. Crack bridging occurs when fibrous materials such as carbon fibers are present inside ceramics, known as carbon fiber-reinforced ceramics [94,95]. Crack propagation is suppressed by the bridging of the fibrous materials across a crack, resulting in higher fracture toughness. Transformation toughening occurs when stress-induced transformations of the metastable phase occur in the vicinity of a propagating crack, such as the tetragonal-to-monoclinic transformation of zirconia [82]. Phase transformation around a propagating crack causes compressive stress around the crack tip due to the local expansion associated with the phase transformation, such as in zirconia and zirconia-containing ceramics [82]. This suppresses the crack propagation, and the fracture toughness increases. As discussed in the next subsection, the toughening mechanism of pre-existing dislocations is different to the above mechanisms.

### 3.2. Toughening Mechanism by Pre-Existing Dislocations

**Figure 9:**
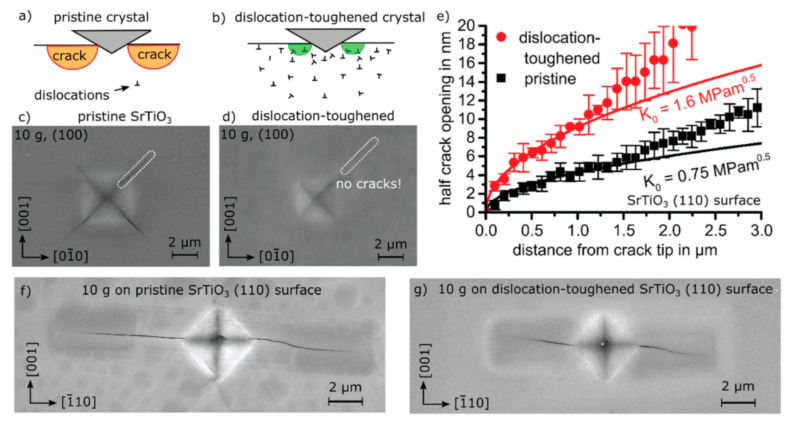
Effect of dislocations on fracture toughness in SrTiO_3_. (**a**) Sketch of Vickers indents inducing cracks on the surface of a pristine crystal and (**b**) in a region with high dislocation density where crack formation can either be completely suppressed or the crack length strongly reduced. (**c**) Vickers indent on a pristine SrTiO_3_ single crystal (100) surface revealing distinct crack formation. (**d**) Same indent on a dislocation-toughened SrTiO_3_ single crystal surface displaying no cracks. (**e**) Crack tip opening displacement on the dislocation-toughened (110) surface indicating a doubling in crack-tip toughness. (**f**) Vickers indent on a pristine SrTiO_3_ (110) surface and (**g**) a (110) surface after dislocation-inducing polishing. Reprinted with permission from Ref. [19]. Copyright 2021, The Royal Society of Chemistry.

The experimental evidence for the toughening of ceramics with pre-existing dislocations is shown in Figure 9 [19]. In the experiment [19], dislocations were introduced in a surface layer of SrTiO_3_ single-crystal ceramics by simple polishing [51]. The depth of the surface layer was about 5 µm, and the introduced dislocation density was about $5\times 10^{14}$ $m^{-2}$, which was quantified using electron channeling contrast imaging (ECCI) in a scanning electron microscope (SEM). Vickers indentation on the dislocation-toughened crystal resulted in no crack formation (Figure 9d), while that on a pristine crystal resulted in distinct crack formation (Figure 9c) [19]. The crack opening profile after Vickers indentation of 0.1 N load, shown in Figure 9e, indicated that the crack-tip toughness, which is slightly below actual fracture toughness, of a dislocation-toughened crystal is about two times higher than that of a pristine crystal [19]. The crack-tip toughness ($K_{0}$) is obtained from the crack opening profile using the following equation [19,96,97].

(23) $$u=K_{0}\times \frac{1-\nu^{2}}{E}\times \sqrt{\frac{8\xi }{\pi }}$$

where u is the half-opening of a crack, and ξ is the distance from the crack tip along the center line of the crack.

There are some other experimental reports [20,21] on dislocation toughening in single-crystal ceramics. Preuβ et al. [20] reported that the fracture toughness of KNbO_3_ was increased by a factor of 2.8 by introducing dislocations into the single crystal up to a density of $10^{14}$ $m^{-2}$ by the cyclic Brinell indentation method [52]. In ref [20], the fracture toughness was estimated from the measurement of the crack-tip opening profile. The dislocation density was estimated from the images using ECCI and PFM (piezo-response force microscopy) [20]. Salem et al. [21] reported that thermal treatment of a SrTiO_3_ single crystal at 1100 °C, which is 58% of the melting point, considerably increased the dislocation density. Before the thermal treatment, dislocations were introduced into the specimen by cyclic Brinell indentation at room temperature. For example, the initial dislocation density of $1\times 10^{13}$ $m^{-2}$ at room temperature was increased to $7\times 10^{13}$ $m^{-2}$ by the thermal treatment at 1100 °C for 10 h [21]. The dislocation density was estimated by the SEM images [21]. By the increase in dislocation density from the thermal treatment, the fracture toughness ($K_{Ic}$) was considerably increased, which was estimated by the indentation crack-length method using a Vickers indenter with the following equation [21,98,99].

(24) $$K_{Ic}=0.035{\left(\frac{a}{l}\right)}^{-0.5}{\left(\frac{E\phi }{H}\right)}^{0.4}\left(\frac{Hl^{0.5}}{\phi }\right)$$

where a is the crack length (from the tip of the indentation imprint to the tip of the crack), l is the half-diagonal of the indentation, E is the elastic modulus, which is 264 GPa for a SrTiO_3_ single crystal at room temperature [100], ϕ=3 [21,98,99], and H is the hardness, which was measured by the Vickers indentation test.

**Figure 10:**
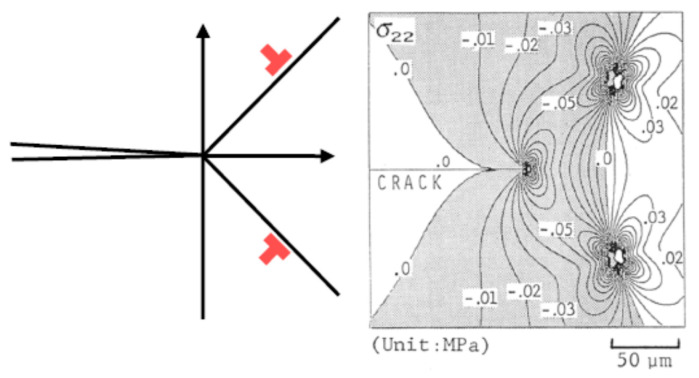
Calculated stress field around a crack tip in the presence of two dislocations indicated by the red symbol. The shaded area indicates compressive stress, which is the crack-tip shielding by dislocations. Reprinted with permission from Ref. [101]. Copyright 2022, The Iron and Steel Institute of Japan.

**Figure 11:**
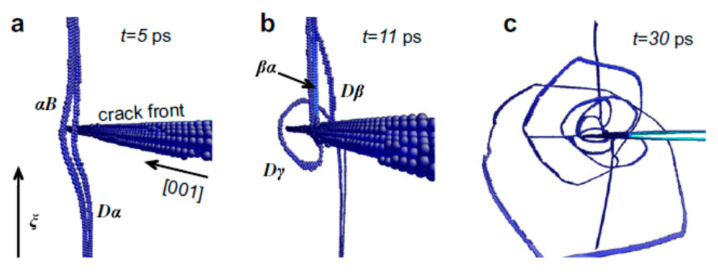
Molecular dynamics simulation of an initially straight dislocation in front of the crack tip. (**a**) The curved dislocation during its approach to the crack tip. (**b**) The stimulated emission of a new leading partial dislocation Dγ and the partial dislocation cross-slip Dα → Dβ + bα at the crack tip. (**c**) Emission of additional leading partial dislocations from the crack tip. Reprinted with permission from Ref. [107]. Copyright 2013, Elsevier.

**Figure 12:**
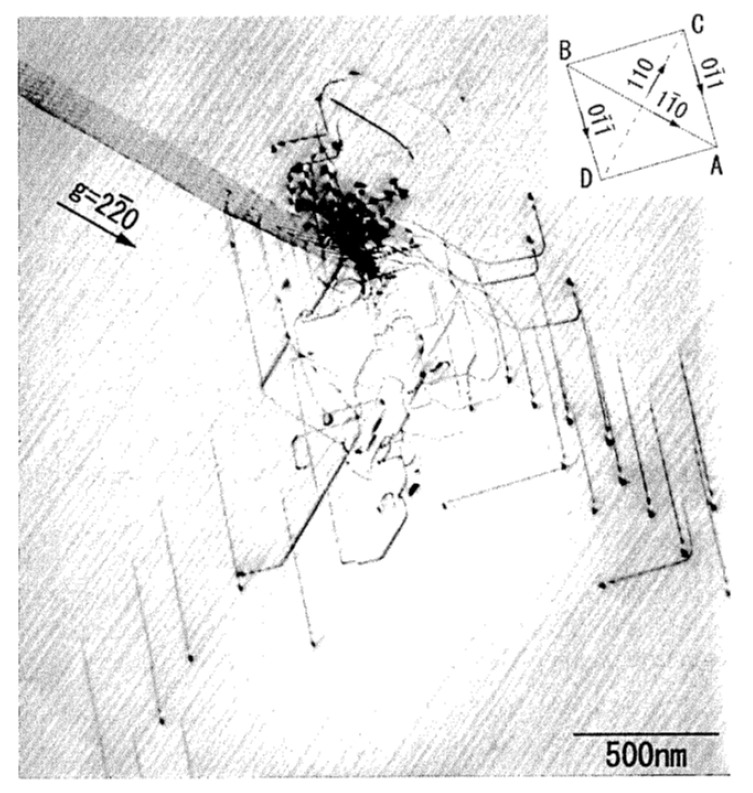
High-voltage electron microscopy (HVEM) image of crack-tip dislocations at the beginning of their multiplication. Reprinted with permission from Ref. [108]. Copyright 2002, Taylor & Francis Ltd.

Next, the mechanism of toughening by pre-existing dislocations is discussed. The presence of dislocations results in some stress fields in the specimen [77,90]. Under some conditions, compressive stress is formed from some dislocations around a crack tip, which suppresses crack propagation and thus increases the fracture toughness (Figure 10) [101]. This is called crack-tip shielding by dislocations [101,102,103,104,105,106]. In the recent experimental reports [19,21] of dislocation toughening, it was suggested that pre-existing dislocations themselves shield the crack tip, resulting in an increase in fracture toughness. However, from many other pre-existing dislocations, tensile stress results around a crack tip, which accelerates crack propagation and thus decreases the fracture toughness [104,105]. This is called crack-tip anti-shielding by dislocations [102,104,105]. In many cases, shielding dislocations are emitted from a crack tip and repelled from it [102,104]. On the other hand, anti-shielding dislocations are emitted from other sources and attracted to a crack tip [102,104]. In other words, pre-existing dislocations themselves do not necessarily shield the crack tip, but dislocations emitted from a crack tip do shield the crack tip [102,104,105]. Thus, for effective crack-tip shielding, dislocations should be emitted from a crack tip. Actually, it has been reported by molecular dynamics simulations that pre-existing dislocations do enhance emissions of dislocations from a crack tip (Figure 11) [107]. For reference, experimental observation of emissions of many dislocations from a crack tip is shown in Figure 12 [108]. In summary, pre-existing dislocations enhance emissions of dislocations from a crack tip, which results in enhanced crack-tip shielding and toughening of ceramics. Furthermore, enhanced emissions of dislocations from a crack tip effectively dissipate energy through local plastic deformation and heating, which further increases the fracture toughness [109,110]. In addition, crack-tip blunting through emissions of dislocations could also suppress crack propagation and increase the fracture toughness [105,106,109,110].

According to Higashida et al. [101], fracture toughness ($K_{Ic}$) in the presence of dislocations near a crack tip is given as follows.

(25) $$K_{Ic}=\sqrt{\frac{2\gamma_{s}E}{1-\nu^{2}}}-k_{D}$$

where $k_{D}$ is the stress intensity factor caused by dislocations and takes a negative value for shielding dislocations. The equation for $k_{D}$ is given in refs [103,111] and the numerical results are shown in Figure 10 [101]. Equation (25) is a modification of Equation (22).

The molecular dynamics simulations shown in Figure 11 were performed for a face-centered cubic (fcc) nickel with a relatively low activation energy for dislocation motion because atomistic simulations are limited to short time periods of the order of nanoseconds [107,112]. The pre-existing straight dislocation on the left side in Figure 11a approaches the crack and parts of the dislocation are strongly curved [107]. Finally, the pre-existing dislocation intersects the crack front, resulting in emissions of multiple dislocations from the crack tip (Figure 11b,c) [107]. It results in shielding of the crack tip and an increase in the fracture toughness. It is suggested that the dynamics of the pre-existing dislocation, the inertia of the dislocation [77,113,114,115,116], and the waves generated upon deceleration of the dislocation by the abrupt reversal of the driving force from strongly attractive to strongly repulsive at the crack tip play an important role in the stimulated dislocation emissions [107]. It has already been suggested that the formation of dislocations near a crack tip is initiated from pre-existing dislocations [117,118,119]. Pre-existing dislocations are attracted to the crack front and form Frank–Read sources of dislocations by undergoing cross-slip near the tip [117,118]. It may be possible that the anti-shielding nature of pre-existing dislocations causes stimulated emissions of dislocations from a crack tip [120].

The emissions of dislocations from a crack tip increase the ductility of the material because the plasticity of a material is caused by emissions and motions of dislocations [82,121]. The phenomenon is strongly related to the brittle–ductile transition (BDT) of materials [118,121,122]. Silicon is brittle at room temperature with a low fracture toughness of about 0.9 MPa·$m^{1/2}$ [122]. At temperatures above about $0.6T_{m}\;$ where $T_{m}\;$is the melting point, Si crystals may be plastically deformed at reasonable strain rates, similarly to fcc metals, resulting in much higher fracture toughness [122]. This is an example of BDT. The mechanism for the BDT is the change in the frequency of the dislocation emissions from a crack tip as they determine the ductility of the material.

The high-voltage electron microscopy (HVEM) image of crack-tip dislocations at the beginning of their multiplication in Figure 12 was for a silicon single crystal [108]. A crack was introduced by the Vickers indentation method on the (001) surface of a Si wafer chip at room temperature. The specimen was annealed at 873 K for 5 min to induce dislocation generation around the crack tip under the presence of residual stress due to the indentation [108]. For HVEM observation, the specimen was thinned by a focused ion beam in addition to chemical polishing. The acceleration voltage of HVEM was 1000 kV [108]. In Figure 12, dislocation arrays are seen not only in the direction of the crack propagation but also in the direction perpendicular to the crack propagation [108]. The dislocations gliding away from the crack tip were emitted in the early stage of dislocation emission. They are almost screw dislocations [108]. In the region within 1 µm of the crack tip, curved and tangled dislocations are seen (Figure 12). The multiplication of crack-tip dislocations is caused by the cross-slip of the dislocations in the array, which results in effective crack-tip shielding and the increase in fracture toughness [108].

## 4. Conclusions and Unsolved Problems

All-dislocation-ceramics, which are ceramic solid-electrolytes filled with appropriate dislocations, are expected to have high ionic conductivity and high fracture toughness compared to normal solid electrolytes. Furthermore, dendrite formation could be avoided because the spatial distribution of ionic current density becomes nearly uniform in all-dislocation-ceramics. High ionic conductivity is due to the considerably higher ionic conductivity along dislocations caused by a higher concentration of vacancies compared to that in the bulk. The toughening mechanism of ceramics by pre-existing dislocations is the enhanced emissions of dislocations from a crack tip, which shield the crack tip, dissipate some energy by local plastic deformation and heating, and blunt the crack tip.

Based on the theoretical prediction [17,18], it is required to experimentally produce all-dislocation-ceramics. It is necessary to find out which method is suitable for the production of all-dislocation-ceramics with respect to the introduction of dislocations into ceramics: applying compressive stress, mechanical polishing, cyclic loading of an indenter, irradiation of ultrasonic wave [123,124,125], cold sintering under high pressure [126,127], etc. It is also required to clarify to what degree fracture toughness could be increased by the introduction of dislocations, especially for the case of all-dislocation-ceramics. It may be interesting to try to make all-dislocation-ceramics using the sintering process of particles under high applied pressures such as hot pressing [128], dry pressing at relatively low temperatures [129,130], and cold sintering with a mediate liquid phase [126,127]. All-dislocation-ceramics may be produced experimentally on a trial-and-error basis, measuring dislocation densities using varying experimental conditions.

## Data Availability

Not applicable.

## Nomenclature

a	crack length (m)
A	amplitude of reduction in ionic conductivity by crossing dislocations (S/m)
b	magnitude of the Burgers vector (m)
B	dimensionless factor which depends on specimen geometry
d	distance between neighboring dislocations (m)
$d_{0}$	characteristic diameter of pre-existing microcracks (m)
$d_{c}$	critical diameter of a pre-existing microcrack for fracture (m)
$d_{crack}$	diameter of a pre-existing penny-shaped (circular) microcrack (m)
E	Young’s modulus (Pa)
$E_{el}$	applied electric field (V/m)
G	shear modulus (Pa)
$g\left(D\right)dD$	number concentration of pre-existing microcracks with diameter between D and D+dD ($m^{-3})$
H	hardness of a specimen measured by the Vickers indentation test
$j_{dis}$	ionic current density on a dislocation (A/$m^{2}$)
$j_{other}$	ionic current density on the other area (A/$m^{2}$)
$k_{D}$	stress intensity factor caused by dislocations and takes negative value for shielding dislocations ($\mathrm{Pa}\cdot m^{1/2}$)
$K_{0}$	crack-tip toughness ($\mathrm{Pa}\cdot m^{1/2}$)
$K_{I}$	stress intensity factor ($\mathrm{Pa}\cdot m^{1/2}$)
$K_{Ic}$	fracture toughness ($\mathrm{Pa}\cdot m^{1/2}$)
l	half-diagonal of the indentation (m)
m	shape factor of the Weibull distribution
n	number concentration of pre-existing microcracks ($m^{-3})$
$n_{d}$	density of dislocations ($m^{-2}$)
$n_{d,limit}$	upper limit of the dislocation density ($m^{-2}$)
N	number of pre-existing microcracks in a specimen
$P_{F}$	probability of fracture
r	distance from a crack tip (m)
R	strength ratio
$S_{d}$	cross section of a dislocation pipe ($m^{2}$)
u	half-opening of a crack (m)
V	volume of a specimen ($m^{3}$)
α	numerical factor defined in Equation (9)
$\gamma_{s}$	surface energy of a solid specimen (J/$m^{2}$)
δ	diameter of a dislocation pipe (m)
$\delta_{c}$	critical diameter of a dislocation pipe (m)
$\delta_{core}$	diameter of a dislocation core (m)
θ	angle of parallel straight dislocations relative to the electrode (rad)
$\theta_{r}$	angle defined in Figure 5 (rad)
κ	mean ionic conductivity (S/m)
$\kappa_{b}$	ionic conductivity in the bulk (S/m)
$\kappa_{d}$	ionic conductivity along a dislocation (S/m)
$\kappa_{d⊥}$	ionic conductivity across a dislocation (S/m)
ν	Poisson’s ratio
ξ	distance from a crack tip along the center line of the crack (m)
σ	applied compressive stress (Pa)
$\sigma_{app}$	magnitude of the applied stress on a specimen (Pa)
$\sigma_{c}$	compressive strength (Pa)
$\sigma_{t}$	tensile strength (Pa)
$\sigma_{xx}$	stress component defined in Figure 5 (Pa)
$\sigma_{yy}$	stress component defined in Figure 5 (Pa)
$\tau_{xy}$	stress component defined in Figure 5 (Pa)
ϕ	numerical factor used in Equation (24)

## References

[1] Manthiram A., Yu X., Wang S. (2017). Lithium battery chemistries enabled by solid-state electrolytes. Nat. Rev. Mater..

[2] Sunandana C.S. (2016). Introduction to Solid State Ionics Phenomenology and Applications.

[3] Julien C., Nazri G.-A. (1994). Solid State Batteries: Materials Design and Optimization.

[4] Kotobuki M., Song S., Chen C., Lu L. (2018). Ceramic Electrolytes for All-Solid-State Li Batteries.

[5] Randau S., Weber D.A., Kötz O., Koerver R., Braun P., Weber A., Ivers-Tiffee E., Adermann T., Kulisch J., Zeier W.G. (2020). Benchmarking the performance of all-solid-state lithium batteries. Nat. Energy.

[6] Kataoka K., Nagata H., Akimoto J. (2018). Lithium-ion conducting oxide single crystal as solid electrolyte for advanced lithium battery application. Sci. Rep..

[7] Yang H., Wu N. (2022). Ionic conductivity and ion transport mechanisms of solid-state lithium-ion battery electrolytes: A review. Energy Sci. Eng..

[8] Southall J.P., Hubbard H.V.S.A., Johnston S.F., Rogers V., Davies G.R., McIntyre J.E., Ward I.M. (1996). Ionic conductivity and viscosity correlations in liquid electrolytes for incorporation into PVDF gel electrolytes. Solid State Ionics.

[9] Kato Y., Hori S., Saito T., Suzuki K., Hirayama M., Mitsui A., Yonemura M., Iba H., Kanno R. (2016). High-power all-solid-state batteries using sulfide superionic conductors. Nat. Energy.

[10] Janek J., Zeier W.G. (2016). A solid future for battery development. Nat. Energy.

[11] Sun H., Mei L., Liang J., Zhao Z., Lee C., Fei H., Ding M., Lau J., Li M., Wang C. (2017). Three-dimensional holey-graphene/niobia composite architectures for ultrahigh-rate energy storage. Science.

[12] Famprikis T., Canepa P., Dawson J.A., Islam M.S., Masquelier C. (2019). Fundamentals of inorganic solid-state electrolytes for batteries. Nat. Mater..

[13] Yuan C., Lu W., Xu J. (2021). Unlocking the electrochemical-mechanical coupling behaviors of dendrite growth and crack propagation in all-solid-state batteries. Adv. Energy Mater..

[14] Athanasiou C.E., Jin M.Y., Ramirez C., Padture N.P., Sheldon B.W. (2020). High-toughness inorganic solid electrolytes via the use of reduced graphene oxide. Matter.

[15] Yan G., Malzbender J., Fu S., Gross J.P., Yu S., Eichel R.-A., Schwaiger R. (2021). Fracture behavior of solid electrolyte LATP material based on micro-pillar splitting method. J. Eur. Ceram. Soc..

[16] Ke X., Wang Y., Ren G., Yuan C. (2020). Towards rational mechanical design of inorganic solid electrolytes for all-solid-state lithium ion batteries. Energy Stor. Mater..

[17] Yasui K., Hamamoto K. (2023). Influence of dislocations on ionic conductivity and dendrite formation in solid electrolytes. Phys. Scr..

[18] Yasui K., Hamamoto K. (2023). Theoretical upper limit of dislocation density in slightly-ductile single-crystal ceramics. J. Phys. Condens. Matter.

[19] Porz L., Klomp A.J., Fang X., Li N., Yildirim C., Detlefs C., Bruder E., Höfling M., Rheinheimer W., Patterson E.A. (2021). Dislocation-toughened ceramics. Mater. Horiz..

[20] Preuβ O., Bruder E., Lu W., Zhuo F., Minnert C., Zhang J., Rödel J., Fang X. (2023). Dislocation toughening in single-crystal KNbO_3_. J. Am. Ceram. Soc..

[21] Salem M.N., Ding K., Rödel J., Fang X. (2023). Thermally enhanced dislocation density improves both hardness and fracture toughness in single-crystal SrTiO_3_. J. Am. Ceram. Soc..

[22] Tang X., Lagerlöf K.P.D., Heuer A.H. (2003). Determination of pipe diffusion coefficients in undoped and magnesia-doped sapphire (a-Al_2_O_3_): A study based on annihilation of dislocation dipoles. J. Am. Ceram. Soc..

[23] Otsuka K., Matsunaga K., Nakamura A., Ii S., Kuwabara A., Yamamoto T., Ikuhara Y. (2004). Effects of dislocations on the oxygen ionic conduction in yttria stabilized zirconia. Mater. Trans..

[24] Armstrong M.D., Lan K.-W., Guo Y., Perry N.H. (2021). Dislocation-mediated conductivity in oxides: Progress, challenges, and opportunities. ACS Nano.

[25] Legros M., Dehm G., Arzt E., Balk T.J. (2008). Observation of giant diffusivity along dislocation cores. Science.

[26] Börgers J.M., Kler J., Ran K., Larenz E., Weirich T.E., Dittmann R., De Souza R.A. (2021). Faster diffusion of oxygen along dislocations in (La,Sr)MnO_3+d_ is a space-charge phenomenon. Adv. Funct. Mater..

[27] Sakaguchi I., Yurimoto H., Sueno S. (1992). Impurities dislocation diffusion in single-crystal MgO. Mater. Sci. Eng..

[28] Heitjans P., Indris S. (2003). Diffusion and ionic conduction in nanocrystalline ceramics. J. Phys. Condens. Matter.

[29] Rabier J., Puls M.P. (1985). Atomistic model calculations of pipe-diffusion mechanisms in MgO. Philos. Mag..

[30] Miller K.M., Ingle K.W., Crocker A.G. (1981). A computer simulation study of pipe diffusion in body centred cubic metals. Acta Metall..

[31] Pun G.P.P., Mishin Y. (2009). A molecular dynamics study of self-diffusion in the cores of screw and edge dislocations in aluminum. Acta Mater..

[32] Luther L.C. (1965). Diffusion along dislocations. J. Chem. Phys..

[33] Porz L., Frömling T., Nakamura A., Li N., Maruyama R., Matsunaga K., Gao P., Simons H., Dietz C., Rohnke M. (2021). Conceptual framework for dislocation-modified conductivity in oxide ceramics deconvoluting mesoscopic structure, core, and space charge exemplified for SrTiO_3_. ACS Nano.

[34] Marrocchelli D., Sun L., Yildiz B. (2015). Dislocations in SrTiO3: Easy to reduce but not so fast for oxygen transport. J. Am. Chem. Soc..

[35] Adepalli K.K., Yang J., Maier J., Tuller H.L., Yildiz B. (2017). Tunable oxygen diffusion and electronic conduction in SrTiO_3_ by dislocation-induced space charge fields. Adv. Funct. Mater..

[36] Garbrecht M., Saha B., Schroeder J.L., Hultman L., Sands T.D. (2017). Dislocation-pipe diffusion in nitride superlattices observed in direct atomic resolution. Sci. Rep..

[37] Bhattacharyya A.J., Tarafdar S., Middya T.R. (1997). Effective medium theory for ionic conductivity in polycrystalline solid electrolytes. Solid State Ion..

[38] Ikuhara Y. (2009). Nanowire design by dislocation technology. Prog. Mater. Sci..

[39] Muhammad Q.K., Porz L., Nakamura A., Matsunaga K., Rohnke M., Janek J., Rödel J., Frömling T. (2021). Donor and acceptor-like self-doping by mechanically induced dislocations in bulk TiO_2_. Nano Energy.

[40] Muhammad Q.K., Bishara H., Porz L., Dietz C., Ghidelli M., Dehm G., Frömling T. (2022). Dislocation-mediated electronic conductivity in rutile. Mater. Today Nano.

[41] Li Y., Fang X., Tochigi E., Oshima Y., Hoshino S., Tanaka T., Oguri H., Ogata S., Ikuhara Y., Matsunaga K. (2023). Shedding new light on the dislocation-mediated plasticity in wurtzite ZnO single crystals by photoindentation. J. Mater. Sci. Technol..

[42] Ikuhara Y. (2009). Oxide ceramics with high density dislocations and their properties. Mater. Trans..

[43] Ren P., Höfling M., Koruza J., Lauterbach S., Jiang X., Frömling T., Khatua D.K., Dietz C., Porz L., Ranjan R. (2020). High temperature creep-mediated functionality in polycrystalline barium titanate. J. Am. Ceram. Soc..

[44] Höfling M., Zhou X., Riemer L.M., Bruder E., Liu B., Zhou L., Groszewicz P.B., Zhuo F., Xu B.-X., Durst K. (2021). Control of polarization in bulk ferroelectrics by mechanical dislocation imprint. Science.

[45] Johanning M., Porz L., Dong J., Nakamura A., Li J.-F., Rödel J. (2020). Influence of dislocations on thermal conductivity of strontium titanate. Appl. Phys. Lett..

[46] Kissel M., Porz L., Frömling T., Nakamura A., Rödel J., Alexe M. (2022). Enhanced photoconductivity at dislocations in SrTiO_3_. Adv. Mater..

[47] Hameed S., Pelc D., Anderson Z.W., Klein A., Spieker R.J., Yue L., Das B., Ramberger J., Lukas M., Liu Y. (2022). superconductivity and ferroelectric quantum criticality in plastically deformed strontium titanate. Nat. Mater..

[48] Otsuka K., Kuwabara A., Nakamura A., Yamamoto T., Matsunaga K., Ikuhara Y. (2003). Dislocation-enhanced ionic conductivity of yttria-stabilized zirconia. Appl. Phys. Lett..

[49] Nakamura A., Matsunaga K., Tohma J., Yamamoto T., Ikuhara Y. (2003). Conducting nanowires in insulating ceramics. Nat. Mater..

[50] Porz L., Knez D., Scherer M., Ganschow S., Kothleitner G., Rettenwander D. (2021). Dislocations in ceramic electrolytes for solid-state Li Batteries. Sci. Rep..

[51] Jin L., Guo X., Jia C.L. (2013). TEM study of <110>-type 35.26° dislocations specially induced by polishing of SrTiO_3_ single crystals. Ultramicroscopy.

[52] Okafor C., Ding K., Zhou X., Durst K., Rödel J., Fang X. (2022). Mechanical tailoring of dislocation densities in SrTiO3 at room temperature. J. Am. Ceram. Soc..

[53] Szot K., Rodenbücher C., Bihlmayer G., Speier W., Ishikawa R., Shibata N., Ikuhara Y. (2018). Influence of Dislocations in Transition Metal Oxides on Selected Physical and Chemical Properties. Crystals.

[54] Amelinckx S., Delavignette P., Newkirk J.B., Wernick J.H. (1962). Dislocations in layer structures. Direct Observation of Imperfections in Crystals.

[55] Kasemchainan J., Zekoll S., Jolly D.S., Ning Z., Hartley G.O., Marrow J., Bruce P.G. (2019). Critical stripping current leads to dendrite formation on plating in lithium anode solid electrolyte cells. Nat. Mater..

[56] Li G., Monroe C.W. (2019). Dendrite nucleation in lithium-conductive ceramics. Phys. Chem. Chem. Phys..

[57] Wu B., Wang S., Lochala J., Desrochers D., Liu B., Zhang W., Yang J., Xiao J. (2018). The role of the solid electrolyte interphase layer in preventing Li dendrite growth in solid-state batteries. Energy Environ. Sci..

[58] Porz L., Swamy T., Sheldon B.W., Rettenwander D., Frömling T., Thaman H.L., Berendts S., Uecker R., Carter W.C., Chiang Y.-M. (2017). Mechanism of lithium metal penetration through inorganic solid electrolytes. Adv. Energy Mater..

[59] Shishvan S.S., Fleck N.A., McMeeking R.M., Deshpande V.S. (2020). Growth rate of lithium filaments in ceramic electrolytes. Acta Mater..

[60] Shishvan S.S., Fleck N.A., McMeeking R.M., Deshpande V.S. (2020). Dendrite as climb dislocations in ceramic electrolytes: Initiation of growth. J. Power Sources.

[61] Cheng E.J., Sharafi A., Sakamoto J. (2017). Intergranular Li metal propagation through polycrystalline Li_6.25_Al_0.25_La_3_Zr_2_O_12_ ceramic electrolyte. Electrochim. Acta.

[62] Maier J., Prill S., Reichert B. (1988). Space charge effects in polycrystalline, micropolycrystalline and thin film samples: Application to AgCl and AgBr. Solid State Ion..

[63] Tuller H.L. (2000). Ionic conduction in nanocrystalline materials. Solid State Ion..

[64] Bellino M.G., Lamas D.G., de Reca N.E.W. (2006). Enhanced ionic conductivity in nanostructured, heavily doped ceria ceramics. Adv. Funct. Mater..

[65] Anderson T.L. (2017). Fracture Mechanics.

[66] Shih C.J., Meyers M.A., Nesterenko V.F., Chen S.J. (2000). Damage evolution in dynamic deformation of silicon carbide. Acta Mater..

[67] Dai J., Su H., Wang Z., Xu J., Fu Y., Chen J. (2021). Damage formation mechanisms of sintered silicon carbide during single-diamond grinding. Ceram. Int..

[68] Yasui K. (2023). Critical roles of impurities and imperfections in various phases of materials. Materials.

[69] Cai W., Nix W.D. (2016). Imperfections in Crystalline Solids.

[70] Bailey J.E., Hirsch P.B. (1960). The dislocation distribution, flow stress, and stored energy in cold-worked polycrystalline silver. Philos. Mag..

[71] François D., Pineau A., Zaoui A. (1998). Mechanical Behaviour of Materials, Volume II: Viscoplasticity, Damage, Fracture and Contact Mechanics.

[72] Cai M. (2010). Practical estimates of tensile strength and Hoek-Brown strength parameter *m_i_* of brittle rocks. Rock Mech. Rock Eng..

[73] Sheorey P.R. (1997). Empirical Rock Failure Criteria.

[74] Lee S., Kim S., Hwang B., Lee B.S., Lee C.G. (2002). Effect of carbide distribution on the fracture toughness in the transition temperature region of an SA 508 steel. Acta Mater..

[75] Tanguy B., Besson J., Pineau A. (2003). Comment on “Effect of carbide distribution on the fracture toughness in the transition temperature region of an SA 508 steel”. Scr. Mater..

[76] Pineau A. (2006). Development of the local approach to fracture over the past 25 years: Theory and applications. Int. J. Fract..

[77] Anderson P.M., Hirth J.P., Lothe J. (2017). Theory of Dislocations.

[78] Munz D., Fett T. (1999). Ceramics: Mechanical Properties, Failure Behaviour, Materials Selection.

[79] Chiang Y.-M., Birnie D.P., Kingery W.D. (1997). Physical Ceramics.

[80] Reichel F., Jeurgens L.P.H., Mittemeijer E.J. (2008). The thermodynamic stability of amorphous oxide overgrowths on metals. Acta Mater..

[81] Canepa P., Dawson J.A., Gautam G.S., Statham J.M., Parker S.C., Islam M.S. (2018). Particle morphology and lithium segregation to surfaces of the Li_7_La_3_Zr_2_O_12_ solid electrolyte. Chem. Mater..

[82] Barsoum M.W. (2020). Fundamentals of Ceramics.

[83] Pelleg J. (2013). Mechanical Properties of Materials.

[84] Tada H., Paris P.C., Irwin G.R. (2000). The Stress Analysis of Cracks Handbook.

[85] Murakami Y. (1987). Stress Intensity Factors Handbook, Volume 1 and 2.

[86] Niu H., Niu S., Oganov A.R. (2019). Simple and accurate model of fracture toughness of solids. J. Appl. Phys..

[87] Jackman S.D., Cutler R.A. (2012). Effect of microcracking on ionic conductivity in LATP. J. Power Sources.

[88] Nonemacher J.F., Arinicheva Y., Yan G., Finsterbusch M., Krüger M., Malzbender J. (2020). Fracture toughness of single grains and polycrystalline Li_7_La_3_Zr_2_O_12_ electrolyte material based on a pillar splitting method. J. Eur. Ceram. Soc..

[89] Ghosh S., Tarafder M., Sivaprasad S., Tarafder S. (2010). Experimental and numerical study of ball indentation for evaluation of mechanical properties and fracture toughness of structural steel. Trans. Indian Inst. Met..

[90] Courtney T.H. (2005). Mechanical Behavior of Materials.

[91] Cho D.-H., Kim Y.-W., Kim W. (1997). Strength and fracture toughness of in situ-toughened silicon carbide. J. Mater. Sci..

[92] Furushima R., Nakashima Y., Maruyama Y., Hirao K., Ohji T., Fukushima M. (2023). Artificial intelligence-based determination of fracture toughness and bending strength of silicon nitride ceramics. J. Am. Ceram. Soc..

[93] Rice R.W. (2000). Mechanical Properties of Ceramics and Composites.

[94] Inoue R., Yang J.M., Kakisawa H., Kagawa Y. (2013). Mode I fracture toughness of short carbon fiber-dispersed SiC matrix composite fabricated by melt infiltration process. Ceram. Int..

[95] Du J., Zhang H., Geng Y., Ming W., He W., Ma J., Cao Y., Li X., Liu K. (2019). A review on machining of carbon fiber reinforced ceramic matrix composites. Ceram. Int..

[96] Seidel J., Rödel J. (1997). Measurement of crack tip toughness in alumina as a function of grain size. J. Am. Ceram. Soc..

[97] Knowles K.M. (2017). The plane strain Young’s modulus in cubic materials. J. Elast..

[98] Niihara K., Morena R., Hasselman D.P.H. (1982). Evaluation of *K_IC_* of brittle solids by the indentation method with low crack-to-indent ratios. J. Mater. Sci. Lett..

[99] Niihara K. (1983). A fracture mechanics analysis of indentation-induced Palmqvist crack in ceramics. J. Mater. Sci. Lett..

[100] Patterson E.A., Major M., Donner W., Durst K., Webber K.G., Rödel J. (2016). Temperature-dependent deformation and dislocation density in SrTiO_3_ (001) single crystals. J. Am. Ceram. Soc..

[101] Higashida K., Tanaka M., Sadamatsu S. (2022). Effect of crack-tip shielding by dislocations on fracture toughness—In relation to hydrogen embrittlement. ISIJ Int..

[102] Xu Y.B., Ha K.F., Wang Z.G., Wang X.H., Li J. (1991). Dislocation distribution near the crack tip of I and II modes in bulk aluminum single crystal. J. Appl. Phys..

[103] Narita N., Higashida K., Torii T., Miyaki S. (1989). Crack-tip shielding by dislocations and fracture toughness in NaCl crystals. Mater. Trans..

[104] Ohr S.M. (1987). Antishielding dislocations at a crack tip. Scr. Metall..

[105] Hirsch P.B., Bradt R.C., Brookes C.A., Routbort J.L. (1995). Crack-tip plasticity and quasi-brittle fracture of single crystals. Plastic Deformation of Ceramics.

[106] Ohr S.M. (1985). An electron microscope study of crack tip deformation and its impact on the dislocation theory of fracture. Mater. Sci. Eng..

[107] Bitzek E., Gumbsch P. (2013). Mechanisms of dislocation multiplication at crack tips. Acta Mater..

[108] Higashida K., Narita N., Tanaka M., Morikawa T., Miura Y., Onodera R. (2002). Crack tip dislocations in silicon characterized by high-voltage electron microscopy. Philos. Mag..

[109] Friedel J. (1964). Dislocations.

[110] Brechet Y., Louchet F. (1993). A physical approach to the toughness problem: From thermodynamics to kinetics—I. The homogeneous case. Acta Metall. Mater..

[111] Narita N., Higashida K., Kitano S. (1987). Dislocation distribution around a crack tip and the fracture toughness in NaCl crystals. Scr. Metall..

[112] Mishin Y. (2004). Atomistic modeling of the ɣ and ɣ^′^-phases of the Ni-Al system. Acta Mater..

[113] Bitzek E., Gumbsch P. (2004). Atomistic study of drag, surface and inertial effect on edge dislocations in face-centered cubic metals. Mater. Sci. Eng. A.

[114] Bitzek E., Gumbsch P. (2005). Dynamic aspects of dislocation motion: Atomistic simulations. Mater. Sci. Eng. A.

[115] Sakamoto M. (1991). High-velocity dislocations: Effective mass, effective line tension and multiplication. Philos. Mag. A.

[116] Frank F.C. (1949). On the equations of motion of crystal dislocations. Proc. Phys. Soc. A.

[117] Schoeck G. (1996). The formation of dislocation rings on a crack front. Philos. Mag. A.

[118] Hirsch P.B., Roberts S.G., Samuels J. (1989). The brittle-ductile transition in silicon. II. Interpretation. Proc. R. Soc. Lond. A.

[119] Michot G. (2011). Interaction of a dislocation with a crack tip: From stimulated emission to avalanche generation. Acta Mater..

[120] de Oliveira M.A.L., Michot G. (1998). Three dimensional analysis of the interaction between a crack and a dislocation loop. Acta Mater..

[121] Hirsch P.B., Roberts S.G. (1991). The brittle-ductile transition in silicon. Philos. Mag. A.

[122] Scandian C., Azzouzi H., Maloufi N., Michot G., George A. (1999). Dislocation nucleation and multiplication at crack tips in silicon. Phys. Stat. Sol. A.

[123] Langenecker B. (1966). Ultrasonic treatment of specimens in the electron microscope. Rev. Sci. Instrum..

[124] Westmacott K.H., Langenecker B. (1965). Dislocation structure in ultrasonically irradiated aluminum. Phys. Rev. Lett..

[125] Yasui K., Hamamoto K. (2021). Importance of dislocations in ultrasound-assisted sintering of silver nanoparticles. J. Appl. Phys..

[126] Tsuji K., Fan Z., Bang S.H., Dursun S., Trolier-McKinstry S., Randall C.A. (2022). Cold sintering of the ceramic potassium sodium niobate, (K_0.5_Na_0.5_)NbO_3_, and influences on piezoelectric properties. J. Eur. Ceram. Soc..

[127] Nur K., Zubair M., Gibson J.S.K.-L., Sandlöbes-Haut S., Mayer J., Bram M., Guillon O. (2022). Mechanical properties of cold sintered ZnO investigated by nanoindentation and micro-pillar testing. J. Eur. Ceram. Soc..

[128] Pezzotti G., Kleebe H.-J. (1996). Quantitative characterization of dislocation density in a-SiC crystals after high-pressure sintering in Si_3_N_4_ matrix. J. Mater. Sci. Lett..

[129] Yasui K., Hamamoto K. (2022). Comparison between cold sintering and dry pressing of CaCO_3_ at room temperature by numerical simulations. AIP Adv..

[130] Bouville F., Studart A.R. (2017). Geologically-inspired strong bulk ceramics made with water at room temperature. Nat. Commun..

